# Application of copper-based ovitraps in local houses in West Sumatra, Indonesia: a field test of a simple and affordable larvicide for mosquito control

**DOI:** 10.1186/s41182-016-0007-8

**Published:** 2016-04-10

**Authors:** Mohamad Reza, Cimi Ilmiawati, Hiroyuki Matsuoka

**Affiliations:** Department of Biology, Faculty of Medicine, Andalas University, West Sumatra, Indonesia; Department of Pharmacology, Faculty of Medicine, Andalas University, West Sumatra, Indonesia; Division of Medical Zoology, Jichi Medical University, 1311-1 Yakushiji, Shimotsuke-shi, 329-0498 Japan

**Keywords:** *Aedes spp*., Copper, Larval control, Ovitrap

## Abstract

**Background:**

The application of oviposition traps (ovitraps) is one of the currently available rational methods used in mosquito control campaigns because it eliminates the larval stage. However, the use of current larvicides is hampered by their cost and applicability. Therefore, a more economical and practical alternative is urgently needed. We previously reported that copper in liquid form is a promising candidate due to its potent larvicide properties in a laboratory setting, affordability, and availability.

**Methods:**

In the present study, a field test was performed by randomly placing copper-filled plastic pots with a concentration of 10 ppm in 21 local houses in Painan City, West Sumatra, Indonesia. Three of these pots including a control were placed inside, while another two were placed outside each of the houses.

**Results:**

After 14 days, a large number of dead first and second instar larvae of *Aedes spp*. were observed in the copper-filled pots. Larvae in the control pots were all viable and thriving. Unhatched eggs and pupae were detected in several pots in the copper-treated group but were excluded from the analysis.

**Conclusions:**

Our field data confirmed that copper is a potential larvicide for ovitraps, particularly in under-resourced areas.

## Background

Eradication campaigns for mosquito-borne diseases are of the utmost importance for public health worldwide. Vector-based interventions are considered a more rational and feasible choice than the challenges and expenses associated with discovering cures and vaccines for these diseases. This approach is suitable for under-developed and developing countries with limited financial, technical, and human resources. Over the past century, the dominant methods recommended for vector control have shifted from larval source management (LSM), house screening, and bednets to indoor residual spraying with effective contact pesticides [1–4].

Due to urgent efforts to develop new vector control methods, novel approaches have emerged, for example, toxic nectar baits [5], repellents [6], oviposition traps [7], and genetic modifications to mosquitoes [8]. These approaches need to be combined in order to optimally function in mosquito control. Moreover, the potential of combinations of interventions that include LSM is worth further study [1].

Our recent findings on the ability and properties of copper to kill mosquito larvae [9] and laboratory testing of the effect of liquid copper at a concentration of 10 ppm on three species of mosquito larvae [10] prompted us to perform a field test in a dengue endemic area of West Sumatra, Indonesia. We placed 10 ppm liquid copper in the form of oviposition traps (ovitraps) in 21 houses in four different areas in Painan City, West Sumatra, Indonesia. This field test was intended to validate our proposition that a low concentration of liquid copper is a practical and affordable LSM option for mosquito control/eradication campaigns especially for dengue hemorrhagic disease.

## Methods

### Preparation of copper solutions

CuSO_4_ solutions were used in these experiments at a copper concentration of 10 ppm. This concentration was prepared from a standard solution of 250 mM CuSO_4_ (Wako Pure Chemical Industries, Ltd., Tokyo, Japan).

### Study site and study period

**Fig. 1:**
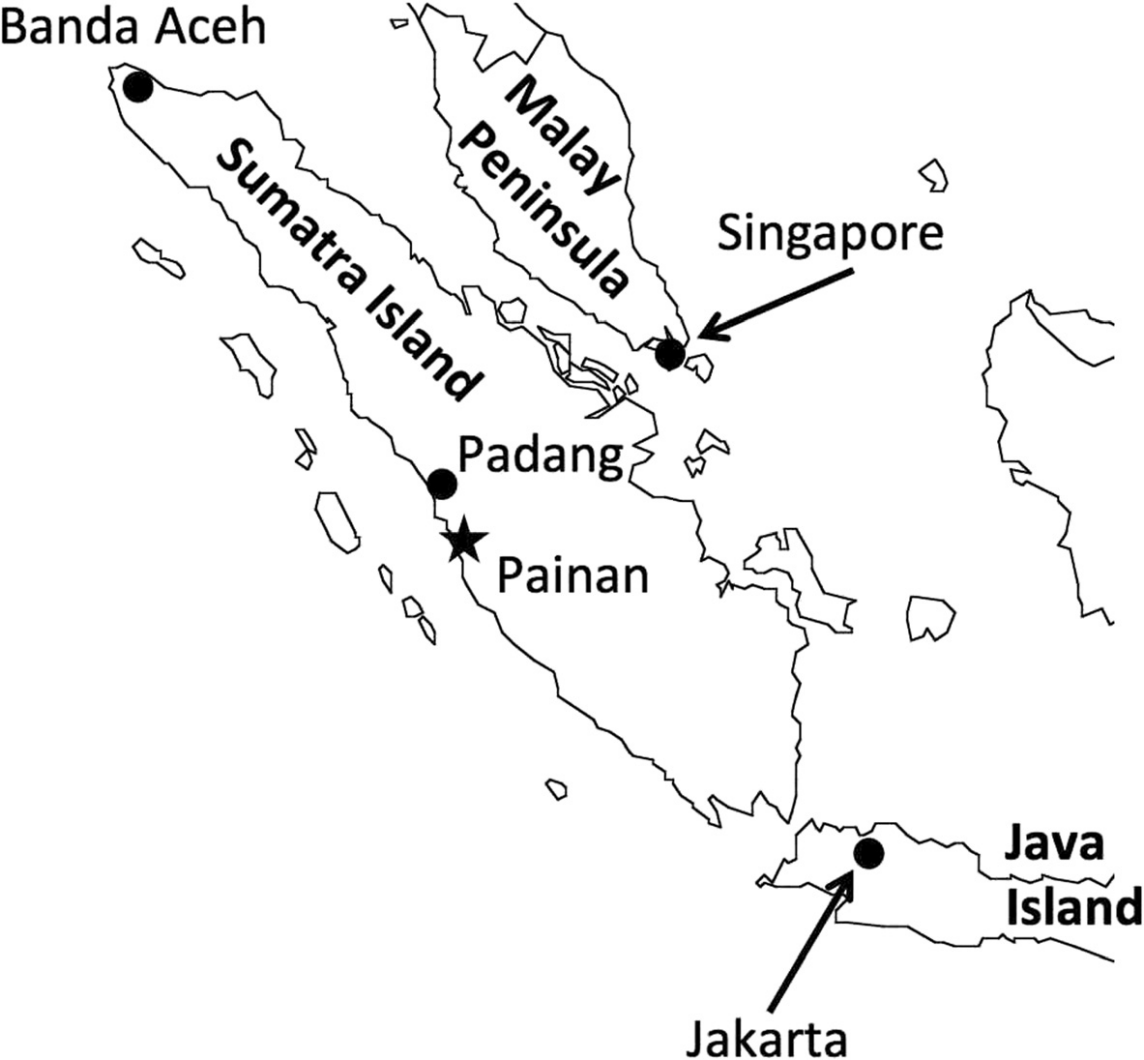
Map of Sumatra Island, Indonesia. Painan City is situated between 1° 05′ south latitude and 100° 30′ east longitude

This study was conducted in Painan City, regency of Pesisir Selatan in the province of West Sumatera, Indonesia (Fig. 1). Painan City faces the Indian Ocean and is endemic for malaria and dengue hemorrhagic fever, with many breeding sites of *Aedes* mosquitoes. Previously, in May 2012 and July 2013, we had investigated adult wild mosquitoes in this area. *Aedes albopictus*, *Aedes aegypti*, and *Anopheles sundaicus* were confirmed to be abundant in this area. The present ovitrap investigation was carried out in July 2015.

### Operation in the field

Twenty-one houses were selected among four designated areas in Painan City with a distance of 100 m between the houses. Five black plastic pots with a diameter of 20 cm and height of 30 cm were prepared for each house and consisted of a water-only filled pot (control) and four copper-treated pots. Each pot was filled with 1 l of tap water, and 630 μl of 250 mM CuSO_4_ was added to the copper-treated pots to reach 10 ppm copper. The pots were prepared directly in the designated house and were distributed in selected areas outside and inside the house. Two copper pots and one control pot were placed inside the house (indoor pots). Two other copper pots were placed in the areas protected or partially protected from the rain and in a 5-m proximity from the house (outdoor pots). The ovitraps were laid for 14 days. During this period, ovitraps were examined every 3 days for maintenance. The numbers of live and dead larvae were counted on day 14. The damaged or spilled ovitraps on the evaluation day were excluded from the experiment. A few pupae (less than five) were observed on day 14 in some control pots. However, they were excluded from the analysis because the number was not recorded. The number of eggs found in ovitraps was also excluded from analysis.

### Statistical analysis

The numbers of viable and dead larvae in each pot were counted, and the difference in dead larvae count between control, inside, and outside pots was analyzed using the Kruskal-Wallis test due to the non-normal distribution of the data, followed by Mann-Whitney U test. Statistical significance was set at *p* value of <0.05. Statistical analyses were performed by MS Office Excel program.

## Results

After 14 days of setting, we found larvae infestation in 70.2 % (66/94) of the pots (Table 1). All were *Aedes spp*. larvae. Among the infestation rates of the control group (65 %), indoor copper group (74 %), and outdoor copper group (69 %), no statistical difference was observed by chi-square test or Fisher’s exact test (Table 2). Mosquitoes laid eggs invariably in copper-treated pots and copper non-treated pots, which suggests that *Aedes* mosquitoes in this area did not avoid the 10 ppm copper solution.

**Table 1:** Larvae distribution in copper treated ovitraps (10 ppm) vs control (0 ppm) placed in 21 houses in Painan City, West Sumatra, Indonesia

House	Control		Indoor pot 1		Indoor pot 2		Outdoor pot 1		Outdoor pot 2	
	Viable	Dead	Viable	Dead	Viable	Dead	Viable	Dead	Viable	Dead
1	1	0	–^a^	–^a^	0	24	0	1	0	3
2	–^a^	–^a^	0	11	0	11	0	0	0	15
3	46	0	0	23	0	5	0	42	0	38
4	38	0	0	23	0	29	0	0	0	0
5	21	0	0	42	0	43	0	15	0	30
6	0	0	0	10	0	0	0	0	0	0
7	77	0	0	35	0	0	40^b^	481	28^b^	500
8	0	0	0	86	0	61	0	6	0	20
9	55	0	0	100	0	53	0	15	0	109
10	–^a^	–^a^	0	0	0	60	0	12	0	22
11	–^a^	–^a^	0	0	–^a^	–^a^	0	63	–^a^	–^a^
12	0	0	0	2	0	5	0	0	0	0
13	0	0	0	300	0	0	0	2	0	15
14	204	0	0	92	0	0	0	84	0	0
15	200	0	0	58	0	109	0	205	0	30
16	55	0	0	408	0	49	0	50	0	80
17	0	0	0	10	0	50	0	150	0	0
18	103	0	0	20	0	0	0	0	0	50
19	–^a^	–^a^	–^a^	–^a^	–^a^	–^a^	–^a^	–^a^	–^a^	–^a^
20	75	0	0	0	0	0	0	120	0	0
21	0	0	0	92	0	0	0	0	0	65
Larva-positive pots (%)	11/17	0/17	0/19	16/19	0/19	12/19	1/20	14/20	1/19	13/19
	(64.7)	(0)	(0)	(84.2)	(0)	(63.2)	(5.0)	(70.0)	(5.3)	(68.4)
Total no. of larvae (mean)	875	0	0	1312	0	499	40	1246	28	977
	(51.3)	(0)	(0)	(69.1)	(0)	(26.3)	(2.0)	(62.3)	(1.5)	(51.4)

^a^Spilled or damaged ovitraps (excluded from analysis)

^b^Larvae appeared weak or dying

**Table 2:** Comparison of the occurrence and number of larvae between control and treatment pots

	Control	Indoor	Outdoor
Proportion of pot with larvae	11/17^a^	28/38^a^	27/39^a^
(Percentage)^c^	(64.7)	(73.7)	(69.2)
Mean number of larvae (live and dead)^d^	51.5^a^	47.7^a^	58.7^a^
(SD)	(65.6)	(81.0)	(119.2)
Mean number of live larvae^e^	51.5^a^	0.0^b^	1.7^b^
(SD)	(65.6)	(0.0)	(7.7)

^a, b^Values with different labels in each row indicate statistically significant difference (*p* < 0.01)

^c^Chi-square test

^d, e^Mann-Whitney U test

Next, we compared the total number of larvae (dead + live) at the 14th day by U test. The mean numbers in each group were statistically no difference (Table 2). However, the larvae mortality rate in copper-treated pots was extremely high (98.3 %). All 875 larvae in the control group were thriving and active. Larvae in the control group varied in number and size, while the copper-treated pots consisted of mostly first and second instar dead larvae. Obviously, control water pots allowed mosquito larvae to survive; however, it was very hard for larvae to survive in the copper-treated pots. Two of the copper-treated containers in no. 7 house had evidence of surviving larvae, albeit in a weak and dying state (Table 1, b).

## Discussion

The result of the present study demonstrated that copper adequately led to a high rate of larval mortality in copper-treated containers. Mortality rates reached 98.3 % in copper-treated pots. In the control pots, all larvae were healthy and active. No dead larvae were found. It may be because the surviving larvae ate dead larvae under the conditions of lacking food. Thus, the survival rate in the control pots (100 %) might be overestimated. However, the lethal effect of the copper is evident as no larvae survived in most of the copper pots. Several surviving larvae were detected in two copper-treated pots outside one house (house no. 7). The house was located near a swamp close to the sea, and the two containers were laid outside a semi-closed bathroom, which allowed rain to dilute the concentration of copper in these pots. We suspect that dilution occurred several days before day 14, because a large number of dead larvae were simultaneously detected with surviving larvae. However, the surviving larvae were small and weak with limited movement. We conclude that although these larvae survived the residual copper concentration, they were still exposed to the damaging effects of copper on their health and development.

The pattern of larval appearance in the pots showed that oviposition occurred without predisposition towards copper-treated and control pots. This result was consistent with our recent findings in which mosquito larvae randomly bred in copper-treated and non-treated environments under laboratory conditions [10]. A statistical analysis with the Mann-Whitney U test on the total number of larvae in each group showed no significant difference. Therefore, we conclude that female mosquitoes breed to equal extent in copper-treated and control ovitraps. It is a limitation of the present study that we did not evaluate the effect of copper solution on egg mortality and hatching success since we did not count hatched and unhatched eggs. Further study is needed to confirm the effect of copper in the egg stage.

Several attractants such as hay and grass infusions [11] may increase the attractiveness of copper ovitraps in the field. A recent study [12] on boric acid-based ovicidal traps reported similar findings to the result of the present study. Another study in Australia also showed a promising result in terms of performance and public acceptance of lethal ovitraps [13]. Our investigation provides evidence that copper may serve as a suitable alternative and/or addition to other ovicidal trap approaches in controlling mosquito populations. Copper ovitraps represent a low-cost, readily available, and easily applicable tool for mosquito control, particularly in under-developed or developing countries with a limited budget and human resources.

A study conducted in Sri Lanka showed that *A. aegypti* and *A. albopictus* have been highly resistant to DDT and have the ability to oviposit indoors and outdoors [14]. Another study in Bangladesh reported abundant potential larval habitats for *Aedes spp*. in containers or jars spread around the city [15]. These situations demand for an inexpensive and simple copper ovitrap in developing countries such as Sri Lanka, Bangladesh, and Indonesia or even in most African countries.

The United States Environmental Protection Agency has limited the concentration of copper to 1 ppm in drinking water. Therefore, although it may be unsuitable to apply copper at a concentration of 10 ppm directly to tap water in order to suppress the number of *Aedes spp*., the use of copper at this concentration in contained water environments such as ovitraps may be a reasonable measure. On the other hand, we need to add copper solution periodically into the ovitraps to keep the copper concentration at 1 to 10 ppm especially during the rainy season. The copper concentration of ovitraps set outside of the house may be flushed and diluted by rain as we experienced in the house no. 7. We need further studies for the maintenance of ovitraps.

## Conclusions

With careful application and strict control from local governments in order to avoid environmental damage due to excessive or irresponsible use of copper, ovitraps may work successfully in the future.
